# Overexpression of miR-150-5p Alleviates Apoptosis in Sepsis-Induced Myocardial Depression

**DOI:** 10.1155/2020/3023186

**Published:** 2020-08-30

**Authors:** Xiao Geng Zhu, Tie Ning Zhang, Ri Wen, Chun Feng Liu

**Affiliations:** Department of Pediatrics, Shengjing Hospital of China Medical University, Shenyang, Liaoning Province 110004, China

## Abstract

Sepsis-induced myocardial depression has high mortality and is very common in intensive care units. Previous studies have found that microRNAs play an important role in regulating sepsis-induced myocardial depression. miR-150-5p is involved in many biological processes; however, the mechanism underlying its role in sepsis-induced myocardial depression is still unclear. In this study, we generated rat models of septic shock induced by lipopolysaccharide. Whole genomic RNA sequencing was performed on 12 left ventricles collected after LPS treatment to identify miRNAs. Most of the target genes of the differently expressed microRNAs were involved in apoptosis, according to Gene Ontology. We also observed apoptosis in the heart tissue and in H9C2 cardiomyocytes stimulated with lipopolysaccharide, indicating that cell apoptosis may be an important mechanism in sepsis-induced myocardial depression. Furthermore, the expression of miR-150-5p was reduced, and overexpression of miR-150-5p with mimics resulted in a decrease in apoptosis, decreased expression of cleaved caspase3 and Bax, and increased expression of Bcl-2. Additionally, after H9C2 cells were transfected with miR-150-5p mimics or an inhibitor, the expression of Akt2 decreased or increased, respectively. These findings suggest that miR-150-5p can alleviate apoptosis and may be a novel therapeutic target for sepsis-induced myocardial depression.

## 1. Introduction

Sepsis is a syndrome of physiological, pathological, and biochemical abnormalities caused by the altered systemic host response to infection [1]. Septic shock is a subset of sepsis in which the underlying circulatory and metabolic abnormalities are profound enough to substantially increase mortality [2]. Heart dysfunction [3] often occurs in septic patients and is referred to as sepsis-induced myocardial depression, which is an important cause of septic shock and increased mortality. It has been reported that myocardial depression occurs in more than 50% of septic patients, and the rate can be as high as 70.2% in pediatric septic patients [4, 5]. However, the specific mechanism underlying sepsis-induced myocardial depression is still unknown.

Previous studies have found that cell apoptosis plays an important role in sepsis-induced myocardial depression [6], but the mechanism which specifically regulates apoptosis in this context is still unclear. Notably, microRNAs (miRNAs) can regulate cell apoptosis. miRNAs are a class of endogenous RNAs that are 19 to 22 nucleotides in length and suppress target gene expression posttranscriptionally in various biological processes [7]. During sepsis-induced myocardial depression, miRNAs may play vital roles in regulating cell apoptosis, and thus influence heart function. For example, in the mouse sepsis model induced by cecal ligation and puncture (CLP), the expression of miR-125b was decreased, apoptosis was alleviated, and cardiac function was improved after transfection of lentivirus expressing miR-125b into the heart [8]. Additionally, in our previous studies, whole genomic sequencing was performed on 12 left ventricles collected from rats with septic shock induced by intraperitoneal injection of lipopolysaccharide (LPS) and control rats [9]. The results showed a differential expression of microRNAs, and the expression of miR-150-5p was decreased in the model group compared to the control group, indicating that miR-150-5p may play a crucial role in sepsis-induced cardiac depression.

We hypothesized that the differentially expressed miR-150-5p is involved in sepsis-induced cardiac depression, and this study is aimed at exploring the functional role of miR-150-5p on apoptosis. Furthermore, we determined the target genes of miR-150-5p using bioinformatics analysis in order to elucidate its regulatory role in cell apoptosis associated with sepsis-induced myocardial depression.

## 2. Materials and Methods

### 2.1. Rat Model of Septic Shock

This study was approved by the ethical committees of Shengjing Hospital of China Medical University (2020PS028K), and all experiments conformed to the relevant regulatory standards. Intraperitoneal injection of LPS was used to establish a rat septic shock model as described previously [10]. Briefly, male adolescent rats (Changsheng Bio, Benxi, China) weighing 170 g to 190 g were anesthetized with 20% urethane (1 g/kg, 1 g urethane was added to 5 mL 0.9% saline). We separated the left femoral artery and connected it to the electrophysiological recorder (Biopac MP150 Biopac Systems, Goleta, CA, USA) to monitor the pressure and injected LPS intraperitoneally to establish the model (20 mg/kg, 10 mg LPS was added to 1 mL 0.9% saline) (Escherichia coli 055: B5, L-2880, Sigma-Aldrich, St. Louis, MO, USA) [2]. Control rats were treated with an equal volume of saline. Septic shock is established when the mean arterial pressure has decreased by 25% to 30%. Samples from the left ventricle were collected and divided into two parts. One part was fixed in 4% paraformaldehyde (PFA) for sectioning, and the other was stored at −80°C for qPCR and western blot.

### 2.2. Cell Culture

H9C2 cardiomyocytes were purchased from the Shanghai Institute of Biochemistry and Cell Biology (Shanghai, China) and were cultured in Dulbecco's Modified Eagle's medium (DMEM, Corning Incorporated, Corning, NY, USA) containing 10% fetal bovine serum (FBS, Biological Industries, Kibbutz Beit Haemek, Israel) and 1% penicillin/streptomycin (Biological Industries) in a humidified atmosphere consisting of 5% CO_2_ and 95% air at 37°C.

### 2.3. Hematoxylin and Eosin (H&E) Staining

The heart samples were fixed in 4% paraformaldehyde (PFA) for 48 hours following dehydration and permeabilization, embedded with paraffin, and sectioned into 4 *μ*m slices. These sections were deparaffinized, stained with hematoxylin for 10 minutes, and then stained with eosin for 1 minute. The sections were dehydrated through a graded alcohol series and observed under a light microscope at 40× magnification.

#### 2.3.1. Terminal Deoxynucleotidyl Transferase dUTP Nick-End Labeling (TUNEL) Assay

H9C2 cardiomyocytes were fixed with 4% formaldehyde for 10 minutes after washing with PBS three times. Next, cells grown on coverslips or heart sections were subjected to the TUNEL assay according to the manufacturer's protocol (Wanlei Bio, Shenyang, China). The apoptotic cells were visualized by fluorescence microscopy.

### 2.4. Cell Viability Detection

H9C2 cells in each group were seeded into 96-well plates (1 × 10^4^ cells per well). After 24 hours, the cells were incubated with different concentrations of LPS (1, 5, or 10 *μ*g/mL) for different amounts of time (12, 24, or 48 hours) [11]. Cell viability was detected using the Cell Counting Kit-8 assay (CCK-8, Dojindo, Kumamoto, Japan) according to the manufacturer's instructions.

### 2.5. Cell Transfection

The miR-150-5p mimics, inhibitor, and respective negative control (NC) were synthesized by GenePharma (Suzhou, China). The mimics, inhibitor, and NC were mixed with JetPrime (PolyPlus, Strasbourg, France) and buffer and incubated for 15 minutes at room temperature. The cells were transfected with the mixture in antibiotic-free medium according to the manufacturer's instructions. Next, the cells were exposed to 10 *μ*g/mL LPS for 24 or 48 hours after transfection. The cells transfected for 24 hours were subjected to RNA extraction, and the cells exposed for 48 hours were used for protein extraction.

### 2.6. Cell Apoptosis Detection

Cells from different groups (control, LPS, LPS+miR-150-5p mimics, and LPS+miR-150-5p NC) were collected after digestion and centrifugation. Cell staining was performed using the Annexin V-FITC/PI Apoptosis Detection Kit (Dojindo, Japan). Cell apoptosis was measured by flow cytometry (BD Biosciences, San Jose, CA, USA) and analyzed using CELL Quest 3.0 software (BD Biosciences).

#### 2.6.1. Quantitative Real-Time Polymerase Chain Reaction (RT-PCR)

Total RNA from the heart samples and H9C2 cardiomyocytes was extracted using RNAiso Plus (TaKaRa, Tokyo, Japan). cDNA was synthesized by reverse transcription with 1 *μ*g total RNA using the PrimeScript™ RT Reagent Kit with gDNA Eraser (Perfect Real Time) (TaKaRa). RT-qPCR was performed using a 7500 Real-Time PCR System (Applied Biosystems, Waltham, MA, USA) according to the TB Green® Premix Ex Taq™ II (Tli RNase H Plus) kit instructions (TaKaRa). GAPDH was used as an endogenous control. The first strand of miR-150-5p was synthesized using the miRNA First Strand cDNA Synthesis Tailing Reaction Kit (Sangon Biotech, Shanghai, China) with 2 *μ*g total RNA. RT-qPCR was performed as mentioned above. U6 was used as an endogenous control. The relative expression levels of mRNA were calculated using the 2^−*ΔΔ*Ct^ method. Primers were all synthesized by Sangon Biotech (Table 1).

### 2.7. Western Blot

The total protein from the tissue and H9C2 cardiomyocytes was extracted using a cell lysis solution (Beyotime Institute of Biotechnology) and phosphatase inhibitor (Meilun Bio, Dalian, USA) and quantified using the Bicinchoninic Acid (BCA) Protein Assay Kit (Beyotime). An equal amount of protein (40 mg/well) was separated by 10% and 12.5% sodium dodecyl sulfate-polyacrylamide gel electrophoresis (SDS-PAGE) and transferred onto polyvinylidene difluoride membranes for 1.5 hours at 100 V. The membrane was then blocked with 5% BSA at room temperature for 1.5 hours. GAPDH, akt2, p-akt2, cleaved caspase3, bax, and bcl-2 primary antibodies were incubated with the membranes at 4°C overnight (Akt2; 1 : 200; Santa Cruz Biotechnology, Santa Cruz, CA, USA; p-Akt2; 1 : 500; Abcam, Cambridge, MA, USA; cleaved caspase3; 1 : 500; Cell Signaling Technology, Danvers, MA, USA; bax and bcl-2; 1 : 500; Proteintech; GAPDH; 1 : 10000; Abways). Subsequently, the membranes were incubated with the appropriate horseradish peroxidase-conjugated secondary antibody (Proteintech) at room temperature for 2 hours. The blotting was visualized using enhanced chemiluminescence (ECL detection kit, KeyGEN Biotech, Jiangsu, China) on the c300 Chemiluminescent Western Blot Imaging System (Azure Biosystems, Dublin, GA, USA).

### 2.8. Bioinformatics Analysis

Based on the sequencing results described previously [9, 12], we selected certain microRNAs for further analysis. The target genes of the differently expressed miRNAs were identified using the miRanda, PITA, and RNAhybrid databases [13–15]. Gene Ontology (GO) and Kyoto Encyclopedia of Genes and Genomes (KEGG) analysis of target genes were performed through The Database for Annotation, Visualization, and Integrated Discovery (DAVID), and statistical significance was set at *P* < 0.05 [16].

### 2.9. Statistical Analysis

The experiments were performed a minimum of three times. All data are presented as mean ± standard deviation (SD). The statistical analyses were performed using GraphPad Prism 8.0 (San Diego, CA). The Student's *t* test for unpaired data was used to compare results between two groups. The one-way analysis of variance (ANOVA) test was used to ascertain significant differences between multiple groups. The two-way ANOVA test was used to ascertain significant differences in the mean arterial pressure (MAP) of the rats between the septic model groups and control groups. Statistical significance was set at *P* < 0.05.

## 3. Results

### 3.1. Septic Shock-Induced Myocardial Apoptosis and Differentially Expressed MicroRNAs in Septic Shock Rats

**Figure 1:**
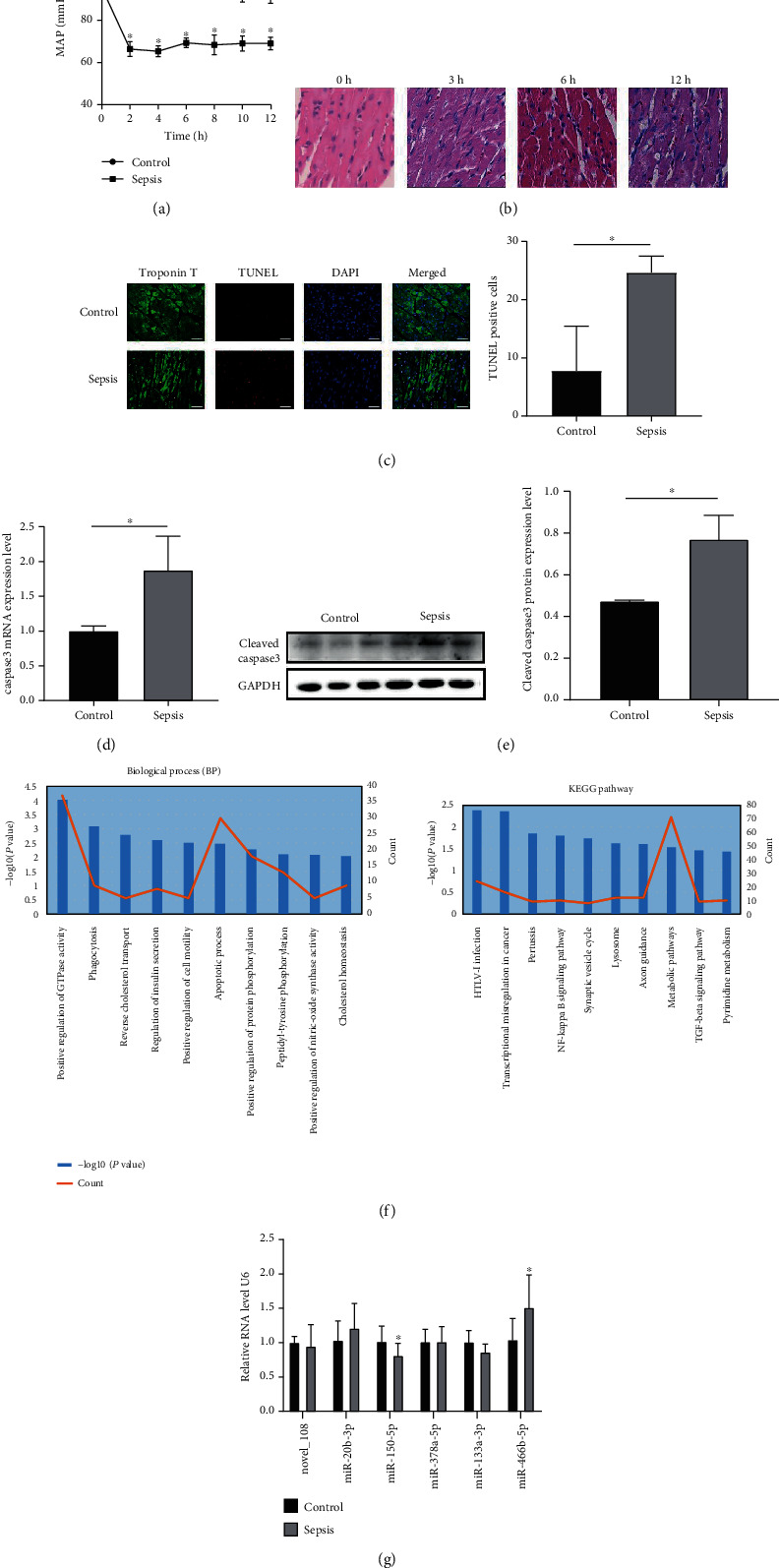
Establishment of the rat septic shock model. (a) Mean arterial pressure of rats from the control and sepsis groups. (b) Pathological changes in rat heart tissues from the control and sepsis groups after LPS injection for 3, 6, or 12 hours by H&E staining. The following results were obtained from rat heart tissues 12 hours post-LPS treatment. (c) Apoptosis was detected using the TUNEL assay. Green represents the cardiomyocytes, red represents the TUNEL positive cells, and blue represents the cell nucleus. (d) The expression of caspase3 mRNA was analyzed by qPCR. (e) The expression of cleaved caspase3 protein was assayed by western blot. (f) The function of target genes of differentially expressed miRNAs from sepsis and control groups was determined by GO enrichment. (g) qPCR validation of selected miRNAs in the rat heart tissues from the control and sepsis groups, *n* = 10. Data are presented as mean ± standard deviation, repeated for three times. ^∗^*P* < 0.05 compared to the control.

As mentioned previously [2, 12], our group has found that there is a positive relationship between the MAP and heart function. In this study, septic shock occurred at approximately 2 hours after LPS injection, and the MAP of the control groups remained unchanged (Figure 1(a)). H&E staining of the left ventricles showed that after 3, 6, and 12 hours of LPS treatment, infiltration of inflammatory cells occurred, there were changes in cell morphology and disordered cell arrangement with delivery time extension (Figure 1(b)), and the injury was most serious at the 12-hour time point. TUNEL-positive cells were increased in the LPS groups compared to the control groups (Figure 1(c)). Additionally, PCR also showed that the expression of caspase3 was increased in the heart of septic shock rats (Figure 1(d)), and similar results were observed when the protein level of cleaved caspase3 was measured (Figure 1(e)).

In the sequencing results mentioned previously [9], 78 miRNAs were expressed differentially in the sepsis and control groups. The results of the GO analysis and KEGG analysis for target genes of differently expressed miRNAs showed that most genes were enriched in the “apoptotic process” (Figure 1(f)). Additionally, we chose some of these miRNAs based on the literature and the relationship between their target genes and apoptosis for further verification in a total of 20 rat left ventricles. As shown in Figure 1(g), the expression of miR-150-5p decreased in the septic shock groups compared with the control groups, indicating that miR-150-5p may exert a crucial influence in sepsis-induced myocardial depression.

### 3.2. LPS-Induced Cell Injury and miR-150-5p Expression in H9C2 Cardiomyocytes

**Figure 2:**
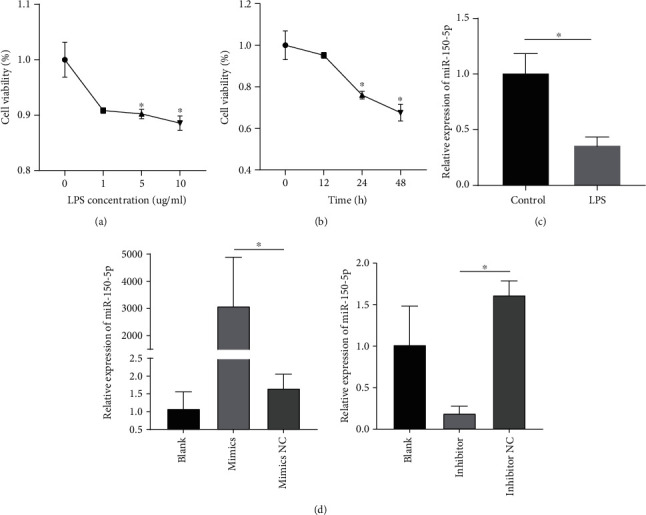
LPS-induced cell injury and miR-150-5p expression in H9c2 cells. (a, b) Cell viability was detected by the CCK-8 assay. (c) The expression of miR-150-5p was measured by qPCR after cells were stimulated with 10 *μ*g/mL LPS for 24 hours. (d) The expression of miR-150-5p after cells was transfected with miR-150-5p mimics or inhibitor. Data are presented as mean ± standard deviation, repeated for three times. ^∗^*P* < 0.05 compared to the control.

As shown in Figures 2(a) and 2(b), the CCK8 assay showed that LPS attenuated cell viability in a dose-dependent manner when cells were treated with different concentrations of LPS (1, 5, or 10 *μ*g/mL) for 24 hours, and cell viability was reduced in a time-dependent manner when the cells were subjected to LPS (10 *μ*g/mL) for 24, 48, and 72 hours. Cardiomyocyte injury was present after exposure to 10 *μ*g/mL LPS for 24 hours. In addition, miR-150-5p expression was suppressed after LPS treatment (Figure 2(c)), which is consistent with the results of the animal experiments. As shown in Figure 2(d), the expression of miR-150-5p decreased or increased when H9C2 cardiomyocytes were transfected with mimics or inhibitor compared to the control, respectively.

### 3.3. miR-150-5p Alleviates H9C2 Cardiomyocyte Apoptosis Induced by LPS Treatment

**Figure 3:**
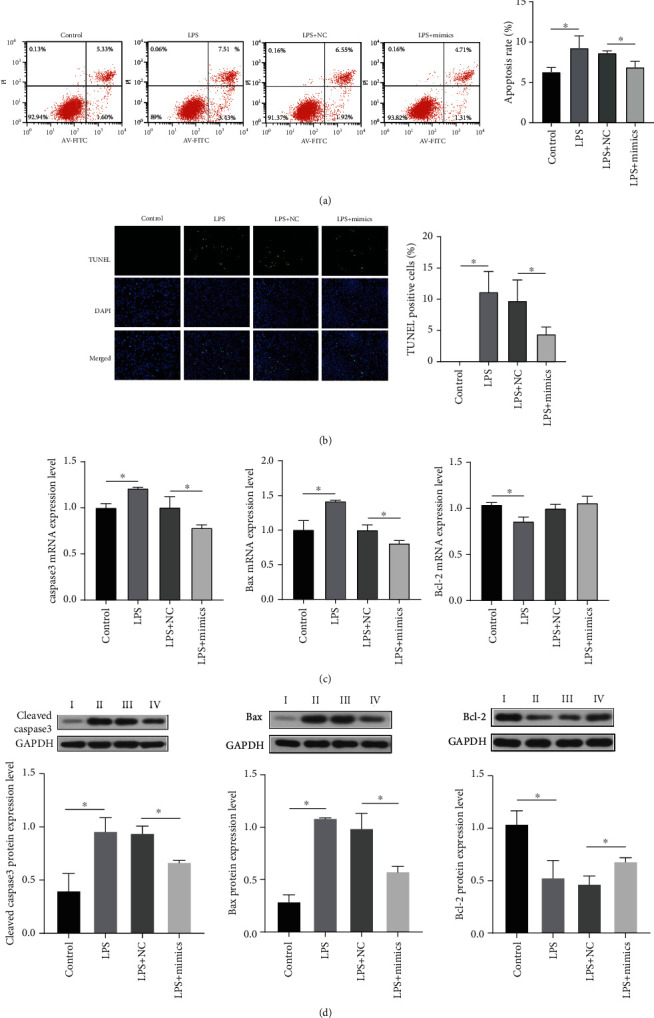
Functional study of miR-150-5p in H9C2 cells. (a, b) Apoptosis was detected by Annexin V-FITC/PI staining (a) and TUNEL assay (b) after transfection. Green represents TUNEL positive cells and blue represents cell nucleus. (a, b) Caspase3, Bax, and Bcl-2 mRNA (c) and protein expression (d) were measured by qPCR and western blot after transfection. I: control, II: LPS, III: LPS+NC, IV: LPS+mimics. Data are presented as mean ± standard deviation, repeated for three times. ^∗^*P* < 0.05 compared to the control or the LPS+NC groups.

To investigate the influence of miR-150-5p on LPS-induced myocardial injury, we evaluated cell apoptosis after overexpression of miR-150-5p. The cells were divided into 4 groups: the control group, LPS group, LPS+NC group, and LPS+mimic group. As illustrated in Figure 3(a), the overexpression of miR-150-5p decreased the rate of apoptosis as measured by flow cytometry, and similar results were seen with TUNEL staining (Figure 3(b)). Additionally, western blot and qPCR analysis demonstrated that the expression of cleaved caspase3 and bax was significantly suppressed at the protein and mRNA levels by miR-150-5p overexpression, while the expression of bcl-2 was increased (Figures 3(c) and 3(d)). These results indicate that miR-150-5p can alleviate apoptosis in LPS-treated H9C2 cardiomyocytes.

**Figure 4:**
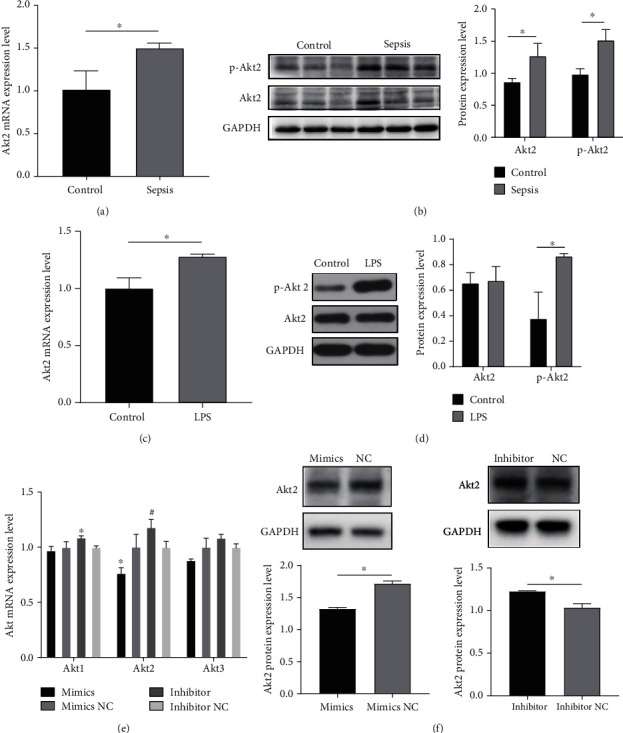
The relationship between miR-150-5p and Akt2. (a, b) Akt2 mRNA (a) and protein (b) were detected in the experimental animals. (c, d) Akt2 mRNA (c) and protein (d) were detected in the H9C2 cells. (e, f) Akt2 mRNA (e) and protein (f) expression were assayed by qPCR and western blot after cell transfection. Data are presented as mean ± standard deviation, repeated for three times. ^∗^*P* < 0.05 compared to the control or the LPS or the NC groups.

Using bioinformatics, we found that there are binding sites between miR-150-5p and the Akt2 3′ untranslated region (UTR), suggesting that Akt2 may be one of the targets of miR-150-5p. Therefore, we further explored the relationship between miR-150-5p and Akt2. The mRNA and protein expressions of Akt2 were increased both in rat heart tissues and H9C2 cells (Figures 4(a)–4(d)) and exhibited the reverse trend of miR-150-5p. In addition, the mRNA (Figure 4(e)) and protein (Figure 4(f)) levels of Akt2 were attenuated after miR-150-5p overexpression in H9c2 cardiomyocytes and elevated after silencing of miR-150-5p. These results demonstrate that miR-150-5p may regulate Akt2 expression. The precise mechanism by which this occurs still needs to be explored in future studies.

## 4. Discussion

Sepsis-induced myocardial depression has received much attention recently due to its high risk of mortality. Because of the high mortality, it is necessary to elucidate the mechanisms underlying its pathogenesis. Numerous studies, including basic research and clinical trials, have confirmed that many regulatory factors play a crucial role in the pathogenesis of sepsis-induced myocardial depression [17, 18]. Apoptosis has been demonstrated to be one of the main causes of decreased heart function [19, 20]. We have observed apoptosis in the cardiomyocytes of septic shock rats and H9C2 cardiomyocytes stimulated with LPS, and we have also confirmed that apoptosis plays a more significant role in sepsis-induced myocardial depression in children [12]. In the present study, we also observed similar results; the apoptotic rate and the expression of cleaved caspase3 protein were increased in LPS-treated rats compared to the control group. These results indicate that cell apoptosis occurs during sepsis-induced myocardial depression, but the specific mechanism by which this occurs is unclear.

Previous studies have demonstrated that miRNAs participate in sepsis-induced myocardial depression in various ways. One is miRNAs regulate target genes expression negatively at posttranscription level. It has been reported that in the rats septic model injected by LPS for 24 h, the expression of miR-146a increased, which inhibited the activation of toll-like receptor 4/nuclear factor kappa-B (TLR-4/NF-*κ*B) signaling pathways and alleviated the cardiomyocytes apoptosis and injury caused by sepsis [21]. The other is that miRNAs involve in the pathogenesis of diseases as a downstream molecules of long noncoding RNAs (lncRNAs). For example, Chen et al. found that p38 mitogen-activated protein kinase (MAPK)/NF-*κ*B, the downstream signaling pathways of miR-125b, was activated and promoted the myocardial injury, which was achieved by the overexpression of lncRNA MALAT1 regulated target miR-125b via competing endogenous RNA (ceRNA) mechanism [22]. In the current study, we found that miR-150-5p expression was decreased in the septic shock rats and H9C2 cardiomyocytes stimulated with LPS compared to the control groups. Furthermore, after the transfection of miR-150-5p mimics or NC, we found that the overexpression of miR-150-5p markedly alleviated the apoptosis, as seen by flow cytometry detection of Annexin V and PI, TUNEL staining, decreased cleaved casapse3 and bax protein and mRNA expression levels, and increased expression of bcl-2. These results suggest that miR-150-5p can alleviate apoptosis in the H9C2 cardiomyocytes stimulated with LPS. In a previous study, a miRNA array of circulating plasma extracellular vesicles from CLP-induced septic mice revealed that the expression of miR-150-5p increased significantly compared to control mice, thus affecting inflammation and the production of inflammatory cytokines and suggesting that miR-150-5p in the myocardial tissue may be differentially expressed [23]. In addition, Yan et al. found that miR-150-5p could inhibit apoptosis by regulating the mutation site V600E of the gene BRAF in papillary thyroid cancer cells [24]. The above findings are consistent with the findings of our current study.

In our study, we found that miR-150-5p could regulate the expression of Akt2. Akt2 belongs to the protein kinase B (PKB, Akt) family, which is mainly expressed in the myocardial tissue and affects cell metabolism, apoptosis, and proliferation. It has been reported that cardiomyocytes deficient in Akt2 are prone to apoptosis [25]. However, other studies have reported that Akt2 deficiency has a protective effect on cardiomyocytes under a high-fat diet and inducible nitric oxide synthase (iNOS) inhibition [26–28]. Besides, Yang et al. also found that the function of IL-6 was inhibited in the mouse with Akt2 knockout, thus alleviating the myocardial injury stimulated by IL-6, which was a protective effect on cardiomyocyte produced by Akt2 inhibition [29]. Although many studies examining Akt signaling pathways in the pathogenesis of sepsis-induced myocardial depression have been carried out [30, 31], most of them were focused on Akt1. As an important molecule of phosphatidylinositol 3-kinase (PI3K)/Akt signaling pathway, Akt1 usually plays a protective role like antiapoptosis or anti-inflammation [31]. However, some studies have pointed out that even if the subtypes of Akt have similar upstream and downstream molecules, they may play a different role in the different diseases and cells [32]. In our study, we found that the Akt2 and p-Akt2 were activated in the LPS-treated rat models and H9C2 cardiomyocytes stimulated by LPS, indicating that Akt2 may be a functional molecule in the pathogenesis in the sepsis-induced myocardial depression, however, it still needs to be determined if Akt2 is a vital regulatory molecule in this context. It has been reported that after Akt2 knockdown, heart function was improved in the LPS-induced mouse sepsis model, which may be achieved by the ubiquitination of Akt2 [33]. Additionally, recent studies have confirmed that glycometabolism is involved in the pathogenesis of sepsis-induced myocardial depression [34], and Akt2 is a crucial molecule involved in glycometabolism [35]. These findings suggest that Akt2 may participate in sepsis-induced myocardial depression via glycosylation.

## 5. Conclusion

This study investigated the role of miR-150-5p in regulating cell apoptosis in sepsis-induced myocardial depression. It has been demonstrated that cell apoptosis is a crucial mechanism of sepsis-induced myocardial depression pathogenesis, and overexpression of miR-150-5p alleviated cell apoptosis in rat myocardial tissues and H9C2 cardiomyocytes treated with LPS. This may represent a novel target for the treatment of myocardial depression caused by sepsis. Although miR-150-5p may regulate the expression of Akt2, further studies are needed to elucidate this mechanism.

## Figures and Tables

**Table 1:** Primer for this study.

	Forward	Reverse
Caspase3	GGAGCTTGGAACGCGAAGAA	ACACAAGCCCATTTCAGGGT
Bax	GCCTTTTTGCTACAGGGTTTCATC	CAATTCGCCTGAGACACTCG
Bcl-2	CTGGTGGACAACATCGCTCT	ATAGTTCCACAAAGGCATCCCAG
Akt1	GGCAGGAGGAGGAGACGATGG	TTCATGGTCACACGGTGCTTGG
Akt2	GCTGGCTGGACTGCTCAAGAAG	TTGATGCTGAGGAAGAACCGATGC
Akt3	ATGATGTGTGGGAGGTTGCC	TGAAGAGAGTGTTCGGGGGAA
GAPDH	GACATGCCGCCTGGAGAAAC	AGCCCAGGATGCCCTTTAGT
U6	GCAAATTCGTGAAGCGTTCCATA	
miR-150-5p	TCTCCCAACCCTTGTACCAGTG	
Universal miRNA primer		AACGAGACGACGACAGAC

## Data Availability

The data used to support the findings of this study are available from the corresponding author upon request.
